# ﻿*Boliviadendron*, a new segregate genus of mimosoid legume (Leguminosae, Caesalpinioideae, mimosoid clade) narrowly endemic to the interior Andean valleys of Bolivia

**DOI:** 10.3897/phytokeys.205.82256

**Published:** 2022-08-22

**Authors:** Élvia Rodrigues de Souza, Priscilla Gomes C. de Almeida, Lamarck Rocha, Erik J.M. Koenen, Margoth Atahuachi Burgos, Gwilym P. Lewis, Colin E. Hughes

**Affiliations:** 1 Universidade Estadual de Feira de Santana, Departamento de Ciências Biológicas, Programa de Pós-graduação em Botânica, Av. Transnordestina s.n., Feira de Santana, Bahia, 44036-900, Brazil Universidade Estadual de Feira de Santana Feira de Santana Brazil; 2 University of Zurich, Department of Systematic and Evolutionary Botany, Zollikerstrasse 107, 8008 Zurich, Switzerland University of Zurich Zürich Switzerland; 3 Present address: Evolutionary Biology & Ecology, Université Libre de Bruxelles, Faculté des Sciences, Campus du Solbosch - CP 160/12, Avenue F.D. Roosevelt, 50, 1050 Bruxelles, Belgium Université Libre de Bruxelles Bruxelles Belgium; 4 Herbario Forestal Nacional Martín Cárdenas, Centro de Biodiversidad y Genética, Universidad Mayor de San Simón, Cochabamba, Bolivia Universidad Mayor de San Simón Cochabamba Bolivia; 5 Accelerated Taxonomy Department, Royal Botanic Gardens, Kew, Richmond, Surrey, TW9 3AE, UK Royal Botanic Gardens, Kew Richmond United Kingdom

**Keywords:** Fabaceae, generic delimitation, *Leucochloron*, monophyly, taxonomy

## Abstract

Phylogenetic analyses of DNA sequence data sampling all species of *Leucochloron* alongside representatives of genera of the Inga and Albizia clades of the larger ingoid clade of mimosoid legumes (sensu Koenen et al. 2020) confirm the non-monophyly of the genus *Leucochloron*. We show that *Leucochloronbolivianum* is placed in the Albizia clade, while the remaining four species of *Leucochloron* are placed in the Inga clade, in line with previous results. To rectify this non-monophyly, *L.bolivianum* is segregated as the new genus, *Boliviadendron*, with a single species, *Boliviadendronbolivianum*, narrowly endemic to the interior Andean valleys of Bolivia. We illustrate this new segregate genus, present a map of its distribution and discuss the striking lack of morphological distinctions between *Boliviadendron* and *Leucochloron*, as well as the phylogenetic and morphological affinities of *Boliviadendron* to the genera *Enterolobium* and *Albizia*.

## ﻿Introduction

The genus *Leucochloron* Barneby & J.W. Grimes was established by Barneby and Grimes (1996) as part of their generic system for the mimosoid legume tribe Ingeae in the New World, with four species, all of them native to Brazil: *L.incuriale* (Vell.) Barneby & J.W. Grimes, *L.limae* Barneby & J.W. Grimes, *L.minarum* (Glaz. ex Harms) Barneby & J.W. Grimes and *L.foederale* (Barneby & J.W. Grimes) Barneby & J.W. Grimes. Barneby and Grimes (1996) based their new genus on the combination of lack of armature, perulate resting buds, homomorphic flowers arranged in globose axillary capitula, broad plano-compressed stiffly papery or weakly coriaceous fruits and shiny discoid exareolate seeds, encircled by a marginal nerve or minute wing.

In May 2002, C.E. Hughes and collaborators collected material of an undescribed mimosoid legume tree from a single locality on the mid-elevation eastern flanks of the Bolivian Andes. A small number of additional collections from nearby localities have been made in subsequent years. This material shares the same combination of characters used by Barneby and Grimes (1996) to delimit *Leucochloron* and, hence, provided the basis for description of a fifth species, *Leucochloronbolivianum* C.E. Hughes & Atahuachi (Hughes and Atahuachi (2006, publ. 2007).

This apparently morphologically commodious generic home for *Leucochloronbolivianum* has been brought into question by phylogenetic evidence from Almeida (2014), LPWG (2017) and more recently generated DNA sequence data for 964 nuclear genes for two species of *Leucochloron*, *L.bolivianum* and *L.limae*, which showed that the genus *Leucochloron* is non-monophyletic (Koenen et al. 2020). Here, we further test this non-monophyly to ascertain where the remaining three species of *Leucochloron* are placed phylogenetically in relation to the two separate *Leucochloron* lineages identified by Koenen et al. (2020), with the aim of providing a solid basis for generic re-delimitation.

## ﻿Materials and methods

Morpho-taxonomic analyses and sample selection for DNA extraction were based on field collections and herbarium specimens from BOLV, FHO, HUEFS, K, LPB, MBM, MEXU, NY, SP and VIC (acronyms follow Thiers 2022, [continuously updated]) collections and previous taxonomic literature (Hughes and Atahuachi 2006, publ. 2007; Almeida 2014).

We sampled 47 accessions (40 of them newly sequenced here and seven using sequences from GenBank) in 19 genera, including all genera of the Albizia and Inga clades sensu Koenen et al. (2020), except *Abarema* s.s., plus a representative set of other lineages spanning the ingoid clade (see Suppl. material 1: Table S1, for GenBank accession numbers and voucher information). All five recognised species of *Leucochloron* were sampled, including seven accessions of *Leucochloronbolivianum*. *Acacia* Mill. (i.e. *Acacia* sensu stricto), which is also a member of the wider ingoid clade, was selected as outgroup.

Total genomic DNA was extracted from silica-dried or herbarium leaf tissue using a modified 2 × CTAB protocol (Doyle and Doyle 1987). DNA samples with low amplification were purified using Sepharose CL-6B (Sigma, St. Louis, Missouri, USA), following the manufacturer’s protocol. We sequenced eight loci which have been used in previous phylogenetic studies of generic relationships in the ingoid clade (e.g., Souza et al. 2013): the nuclear ribosomal 5.8S subunit and flanking ITS1 and ITS2 spacers, the external transcribed spacer ETS and the plastid loci *rps16*, *psbA-trnH*, *rpL32-trnL*, *trnL intron*, *trnL-F* and *trnD-T* (see Suppl. material 1: Table S2 for primer sequences and amplification protocols). The ITS amplifications were performed with primers 17SE and 26SE or primers ITS92 and ITS4. The *trnD-T* region was amplified in two independent reactions combining primers: *trnD^guc^* + *trnE^uuc^* and *trnY^gua^* + *trnT^ggu^* (Shaw et al. 2007).

Polymerase Chain Reactions (PCR) were performed using TopTaq Master Mix Kit (QIAGEN GmbH, Hilden, Germany) according to the manufacturer’s protocol, in 10–15 μl of final reaction volume. For the nrITS and ETS amplifications, 1.0 M betaine and 2% DMSO (dimethylsulphoxide; 2% of the preparation volume) were added. PCR products were purified by enzymatic treatments with Exonuclease I and Shrimp alkaline phosphatase (kit ExoSapIT, GE Healthcare Buckinghamshire, U.K.). Sequencing reactions were performed using the same primers used for PCR and Big Dye Terminator kit v.3.1 (Applied Biosystems, Foster City, California, U.S.A.), on the ABI3130XL Analyzer (Applied BioSystems), at the Laboratório de Sistemática Molecular de Plantas (LAMOL), of the Universidade Estadual de Feira de Santana (UEFS), Bahia, Brazil.

Electropherograms were assembled in Geneious 5.3.6 (Drummond et al. 2010) using default assembly settings. Consensus sequences were aligned in MUSCLE (Edgar 2004) using Geneious, with manual adjustments. We analysed three datasets: 1) the combined plastid loci, 2) combined nuclear loci and 3) combined plastid + nuclear loci. Incongruence between datasets was assessed via 1,000 replicates of the Homogeneity of Participation Test (Felsenstein 1985) using PAUP v. 4.0b10 for Windows (Swofford 2002).

Maximum Parsimony (MP) analyses were performed using PAUP v.4.0 (Swofford 2002), using Fitch parsimony (all characters unordered and equally weighted; Fitch 1971), 1,000 replications of taxon addition and resampling by tree bisection-reconnection (TBR) branch swapping, saving up to 15 trees per replication to prevent extensive searches in sub-optimal islands. Trees from the first search were used as a starting point for a second search using the same parameters retaining a maximum of 10,000 trees. Non-parametric bootstrapping (BS; Felsenstein 1985) was used to estimate clade support, assessed through 1,000 replications, simple taxon-addition and the TBR algorithm, saving up to 15 trees per replicate.

Maximum Likelihood (ML) analyses were performed using RAxML v.8 (Stamatakis 2014), on the CIPRES Science Gateway v.3.3 (Miller et al. 2010), using the GTR + CAT evolutionary model and estimating GAMMA distribution during the run. Branch support was evaluated using 1,000 rapid bootstrap replicates.

Best-fitting substitution models for each data partition were selected using the Akaike Information Criterion (AIC) using MrModeltest v.2.3 (Nylander 2004) (Suppl. material 1: Table S2). Bayesian Inference (BI) analysis was performed on MrBayes v.3.2.6 (Ronquist and Huelsenbeck 2003; Ronquist et al. 2012), on the CIPRES Science Gateway v.3.3 (Miller et al. 2010). Two separate runs of a Metropolis-coupled Markov Chain Monte Carlo (MCMC) were initiated with random starting trees and four simultaneous chains set at default temperatures (Ronquist and Huelsenbeck 2003) for 30 million generations, sampling once every 10^3^ generations. Convergence was assessed using Tracer v.1.6 (Rambaut et al. 2014) to ensure effective sample sizes (ESS) ≥ 200. MrBayes was also used to summarise 75% post burn-in trees sampled from a 50% majority rule consensus tree that included posterior probabilities (PP) as branch support estimates. All trees from MP, BI and ML were visualised and edited in FigTree 1.4.4 (Rambaut 2018).

## ﻿Results

**Figure 1.**
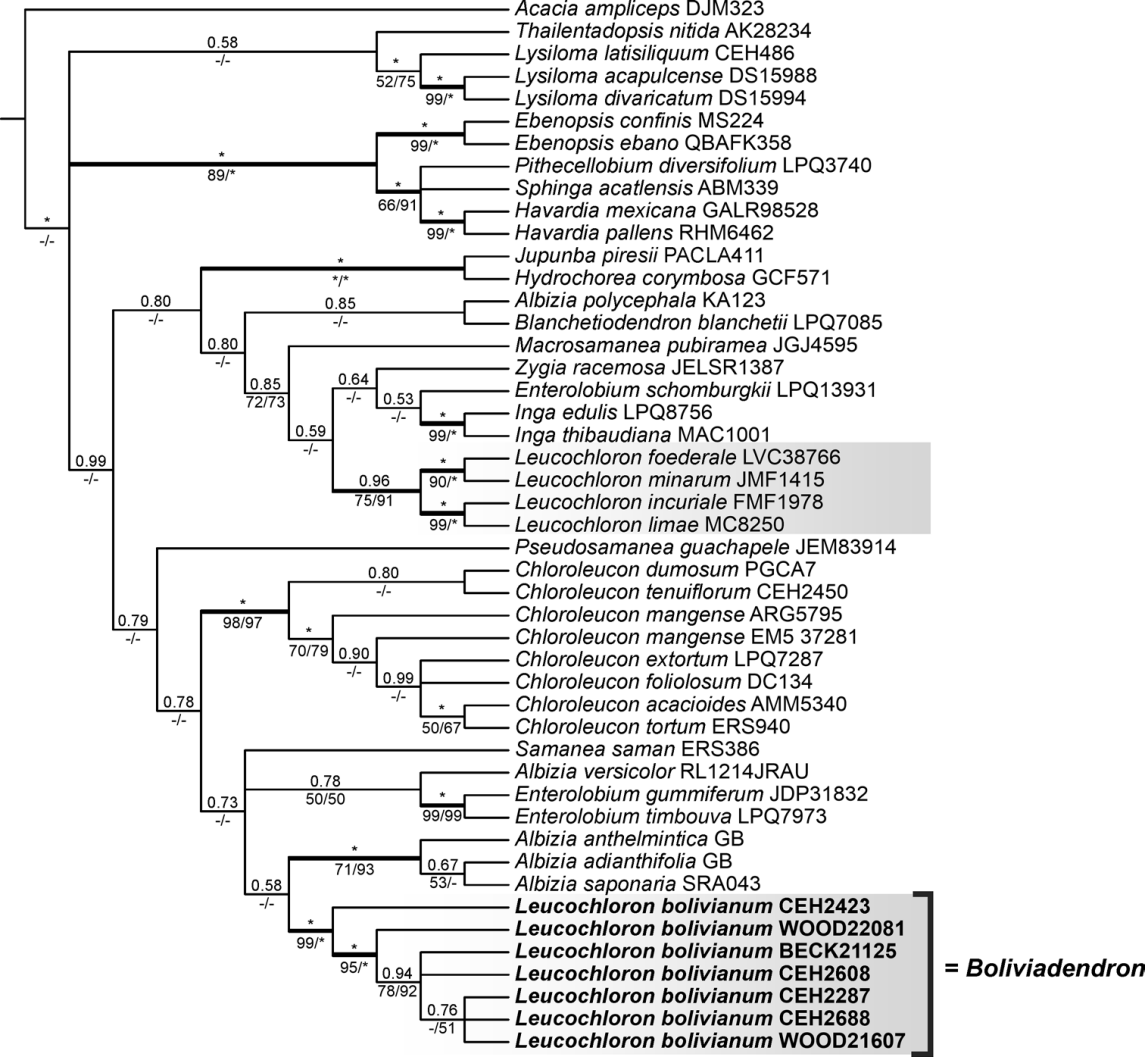
Majority-rule (50%) consensus tree from Bayesian analysis of the combined nuclear (ITS, ETS) and plastid (*psbA-trnH*, *rpL32*, *rps16*, *trnD-T*, *trnL-F*) data for *Boliviadendron* and related genera. Values above branches are Bayesian Posterior Probabilities (PP), below are Bootstrap Support (BS) percentages from the maximum parsimony (left) and Maximum Likelihood analyses (right). Branches supported by PP ≥ 95% are in bold. Only BS values ≥ 50% are shown; - indicates BS ≤ 50%; * indicates PP = 1.0 or BS = 100%.

While separate analyses of the nuclear ITS + ETS and plastid loci yielded gene trees that were, in many cases, poorly resolved (Suppl. material 1: Table S3, Suppl. material 2: Figs S1–S4), the combined nuclear + plastid analysis (Fig. 1) is sufficiently resolved to demonstrate that the genus *Leucochloron* is not monophyletic, with all seven accessions of *L.bolivianum* forming a robustly supported clade placed in a clade with *Albizia* Durazz., *Enterolobium* Mart. and *Samanea* (Benth.) Merr. and the other four species of *Leucochloron* forming a robustly supported clade that is placed in a clade with *Inga* Mill., *Zygia* P. Browne and *Macrosamanea* Britton & Rose ex Britton & Killip. (Fig. 1).

## ﻿Discussion

The non-monophyly of the genus *Leucochloron* is supported by two independent phylogenetic studies, one presented here sampling all five species currently assigned to the genus, based on a small set of widely-used nrDNA and plastid DNA sequence loci (Fig. 1) and the other sampling just two species, but based on phylogenomic analyses of 964 nuclear gene sequences and a broad sample of genera across the whole mimosoid clade (Koenen et al. 2020). This non-monophyly is further confirmed in a more recent phylogeny constructed using a slightly expanded set of 997 nuclear genes, derived from a modified version of the *Mimobaits* gene set of Koenen et al. (2020), which sampled all but five of the 151 genera of subfamily Caesalpinioideae (Ringelberg et al. 2022). This thorough sampling of both taxa and genes demonstrates beyond doubt that *Leucochloron* is indeed non-monophyletic, with *L.bolivianum* placed in the Albizia clade (sensu Koenen et al. 2020) and the remaining four species of *Leucochloron* forming a robustly-supported clade in the Inga clade (sensu Koenen et al. 2020; Fig. 1). These results provide strong evidence supporting segregation of *L.bolivianum* as a new genus, here named *Boliviadendron*.

This robustly-supported non-monophyly is unexpected, given the close morphological similarities between *L.bolivianum* and the other four species of *Leucochloron*. Indeed, there appear to be very few qualitative morphological differences separating *Boliviadendron* (i.e., *L.bolivianum*) from *Leucochloron*, suggesting that the original combination of character states used by Barneby and Grimes (1996) to delimit *Leucochloron* has essentially been recapitulated in *Boliviadendron*. These striking morphological similarities between *Leucochloron* and *Boliviadendron* must, thus, be viewed as further evidence of the extensive morphological homoplasy evident across mimosoids which has frequently misled generic delimitation (Ringelberg et al. 2022). For example, the presence of minute wing-like rims of seeds, which was one of the characters supporting placement of *L.bolivianum* in the genus *Leucochloron* (Hughes and Atahuachi 2006, publ. 2007), have been documented across phylogenetically scattered mimosoid legume lineages, including the genera *Mimozyganthus* Burkart (Luckow et al. 2005), *Archidendropsis* I.C. Nielsen and *Leucochloron* and now here in the phylogenetically distinct lineage *Boliviadendron*. This suggests that this character, just like seeds with true wings which also occur across a disparate set of unrelated mimosoid genera, is homoplasious and evolutionarily labile.

Morphological characters which weakly separate *Boliviadendron* from *Leucochloron* s.s. are: (i) the leaflet base is more evidently asymmetrical in *Boliviadendron* than in the species of *Leucochloron* and the under-surface of *Boliviadendron* leaflets has 1–2 (–3) prominent primary veins, but otherwise the venation is not evident vs. the evident reticulate secondary and tertiary venation on the lower leaflet surface in *Leucochloron* species; (ii) in *Boliviadendron*, the upper leaflet surfaces are consistently blotched purple-black, while leaflets of *Leucochloron* s.s. species are strongly discolorous, with the upper surface drying dark brown and often glossy; (iii) the indumentum in *Boliviadendron* tends to be shorter and white, especially on the corollas and calyces which have fine white, appressed, silky trichomes vs. the generally more ferruginous and longer indumentum, occasionally with golden and/or white hairs intermixed, of *Leucochloron* species; (iv) the number of pollen grains per polyad is constant in *Boliviadendron* at 16 (counted on the isotype *Hughes 2423* and *Wood 21618* at K), while in *Leucochloron* s.s., it is variable (even within species) with 16, 18, 24 or 32 grains.

In the Koenen et al. (2020) phylogeny, *Boliviadendron* appeared as sister to a clade comprising *Enterolobium* and *Albizia* s.s. (i.e., Old World *Albizia*). In the phylogeny presented here, *Boliviadendron* is also placed in a clade with *Enterolobium* and *Albizia* s.s. (Fig. 1) in line with the findings of Koenen et al. (2020). This close relationship of *Boliviadendron* to *Enterolobium* is reflected in the closely similar leaf and flower morphologies of these two genera, including the asymmetrically displaced primary vein of the leaflets (which also characterises many species of *Albizia* s.s.), the blotched purple-black upper leaflet surfaces and superficially very similar globose axillary capitula of homomorphic flowers immersed amongst coevally developing leaves. However, in fruit, these two genera are immediately distinguishable by the characteristically thickened indehiscent curved ‘ear-pod’ fruits of *Enterolobium* vs. the plano-compressed, inertly dehiscent fruits with chartaceous valves of *Boliviadendron*. This means that material of *Boliviadendron* lacking fruits can be difficult to distinguish from *Enterolobium*. Indeed, one of the collections, cited as *L.bolivianum* by Hughes and Atahuachi (2006, publ. 2007) (*J.R.I. Wood 22134* at K, which has leaves and flowers only), has since been correctly identified as *Enterolobiumcontortisiliquum* (Vell.) Morong. Closer examination of the inflorescences of these two taxa, which can occur in close geographical proximity in Bolivia, shows that the flowers of *Boliviadendron* are essentially sessile, while those of *Enterolobiumcontortisiliquum* are pedicellate, on more robust inflorescences. The plano-compressed fruits with stiffly papery chartaceous or weakly coriaceous valves dehiscing inertly along both sutures in *Boliviadendron* are strongly reminiscent of the fruits of the other closely related genus, *Albizia* s.s., but the marked lack of a pleurogram on the seeds in *Boliviadendron* clearly distinguishes the genus from *Albizia*. It is notable that polyads, like those of *Boliviadendron*, are also 16-celled in *Albizia* s.s. (although only few species of the genus have been studied for their pollen characteristics), but can be 16 or 32-celled in *Enterolobium*.

## ﻿Taxonomy

### ﻿ Boliviadendron

Taxon classificationPlantae FabalesLeguminosae

E.R. Souza & C.E. Hughes gen. nov.

6F760332-0B1A-5B60-BEBC-B51613BF5AE7

urn:lsid:ipni.org:names:77303834-1

Fig. 2

#### Type.

*Boliviadendronbolivianum* (C.E. Hughes & Atahuachi) E.R. Souza & C.E. Hughes.

#### Diagnosis.

*Boliviadendron* is similar in almost all respects to the genus *Leucochloron* and is segregated first and foremost because these two lineages are phylogenetically not closely related (Fig. 1; Koenen et al. 2020). *Boliviadendron* differs from *Leucochloron* in its more evidently asymmetrical leaflet bases, by having just 1–2 (–3) primary veins visible vs. the evident reticulate secondary and tertiary venation on the lower leaflet surface in *Leucochloron*, by the consistent purple-black blotches on the upper surface of the leaflets (sometimes the whole surface suffused purplish-black, but never glossy) vs. the strongly discolorous leaflets of *Leucochloron* where the upper surface dries dark brown and is often glossy and finally, by the shorter, white indumentum vs. the more generally ferruginous and longer indumentum of *Leucochloron* species.

#### Description.

**Figure 2.**
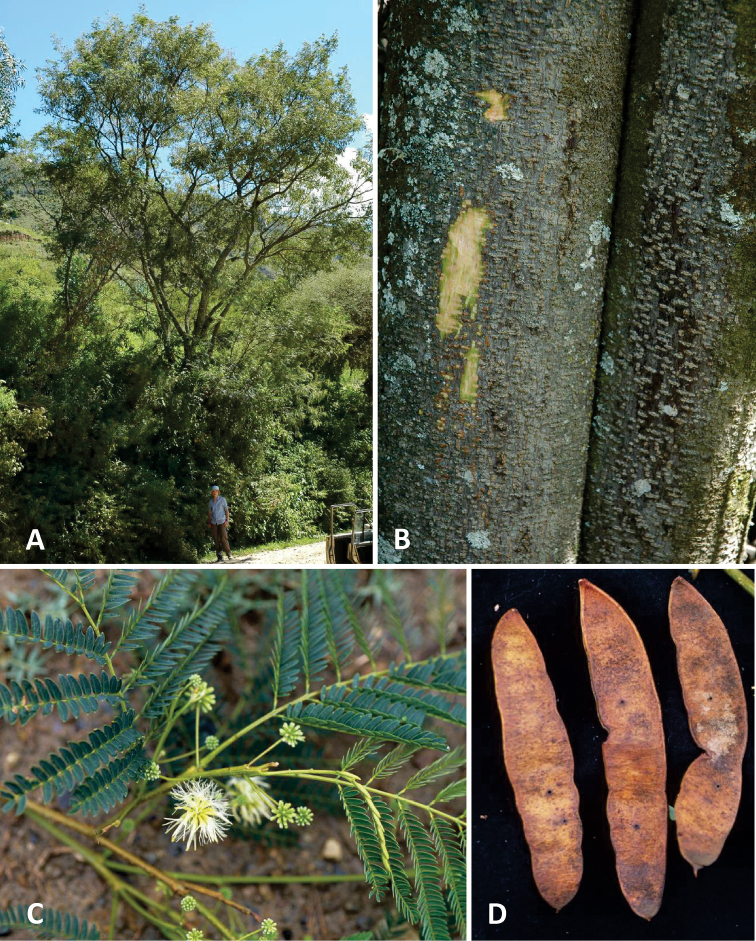
*Boliviadendronbolivianum***A** habit **B** bark and slash **C** flowers and leaves **D** fruits **A, B** from *Hughes et al. 2608***C,D** from *Hughes et al. 2423*. All photos Colin Hughes.

(modified from Hughes and Atahuachi 2006, publ. 2007). Small, unarmed multi-stemmed tree to 5–6 (–10) m tall and 20–35 cm dbh with an irregular spreading crown (Fig. 2A). Outer bark smooth, mid grey-brown, with horizontal lines of lenticels when young (Fig. 2B), becoming thick and corky and vertically fissured with age; inner bark reddish, then cream, gritty. Woody shoots glabrate, lenticellate, dull grey, lenticels round pustulate, the outer bark papery, slightly striate and sometimes slightly exfoliating. Young shoots, leaf stalks and peduncles minutely and sparsely to densely puberulent with whitish or dull golden 0.1– 0.2 mm hairs. **Resting buds** perulate, ovoid to 1.5 mm long and densely pilosulous (as for shoots), but these generally lacking. **Stipules** linear-triangular, 3 × 1.7–2 mm, caducous. **Leaves** bipinnate with 4–5 (–6) pairs of pinnae, leaf stalks canaliculate with two parallel longitudinal ridges, 7–11 cm long, ending in a 2 mm long mucro and including a 2.5–3.6 cm long petiole charged with a 1–1.3 mm diameter circular or weakly ellipsoid sessile or subsessile, thick-rimmed, cupular nectary at or slightly above mid-petiole position; similar smaller nectaries variably present between distal 1–2 pairs of pinnae or sometimes between all pinnae pairs. Longer interpinnal segments 1.3–1.7 cm long, the rachis of longer pinnae 6–8 cm with 15–20 pairs of leaflets per pinna. Leaflets slightly decrescent proximally and distally, larger leaflets 7–10 × 2.1–3.0 (–3.2) mm, not strongly discolorous, nearly glabrous or very sparsely puberulent above, sparsely puberulent below, on small, ridged, transversely elliptic, asymmetric 3 × 0.6 mm pulvinules, the first anterior or first pair of leaflets reduced to 1.2–1.5 mm long paraphyllidia. Leaflets linear-oblong, obliquely truncate at base, obtuse at apex, the tip sometimes acute, venation indistinct, palmate-pinnate brochidodromous, with 1–2 (–weakly 3) primary veins at base, the main primary vein distinctly asymmetric, dividing the blade 1:2–2.5, giving rise on each side to 3–4 sinuous secondary veins, albeit these often indistinct or obscured by the indumentum, venation very faintly immersed or invisible above, the 1–2 (–3) primary veins clearly visible and weakly prominulous below. **Flowers** sessile, homomorphic and arranged in lax globose capitula (Fig. 2C) on 2.5–3 cm-long slender peduncles, moriform in bud, 12–13 mm in diameter (excluding the filaments), the receptacle 1.3 mm in diameter, each capitulum with 20–25 flowers, whitish-green in bud due to dense silky puberulent indumentum on calyx and corolla. The capitula immersed in new foliage, solitary or in fascicles of 2–3 in axils of coeval leaves. Bracts sessile, short spatulate, 0.2–0.4 mm long, puberulent. Calyx narrowly campanulate, 2.5 mm long, the triangular lobes 0.3–0.4 mm long. Corolla tubular, slightly funnel-shaped, 5–6 mm long, the free lobes 1 mm, slightly reflexed. Androecium of ca. 40 stamens to 10 mm long, fused basally into a 3–4 mm long tube that is not or hardly exserted beyond the corolla. Ovary 0.8 mm long, sessile, the style 2.5 mm, held below anthers of the stamens. **Pods** (Fig. 2D) 1 per capitulum, sessile, (6–) 7–10 × 1.7–2 (–2.7) cm, (3–) 4–6 (–8)-seeded, flat plano-compressed, linear-oblong or oblong, obtuse at each end, sometimes with a short apiculum at apex, the valves straight or with slightly undulate margins, stiff papery, pale whitish- or yellow-green when unripe, turning dull orange-brown when ripe, obscurely venulose, sparsely and minutely puberulent overall, internally dull stramineous, framed by plain sutures < 1 mm wide, tardily dehiscent along both sutures, opening narrowly to release seeds, the valves remaining attached after dehiscence. Seeds horizontal at middle of pod, flat disciform, broadly suborbicular, 11–13 mm in diameter, < 1 mm thick, the testa thin, lustrous, translucent castaneous, slightly wrinkled when dry, pleurogram lacking, surrounded by a narrow 0.4–0.6 mm dark marginal nerve or minute wing.

#### Geographic distribution.

**Figure 3.**
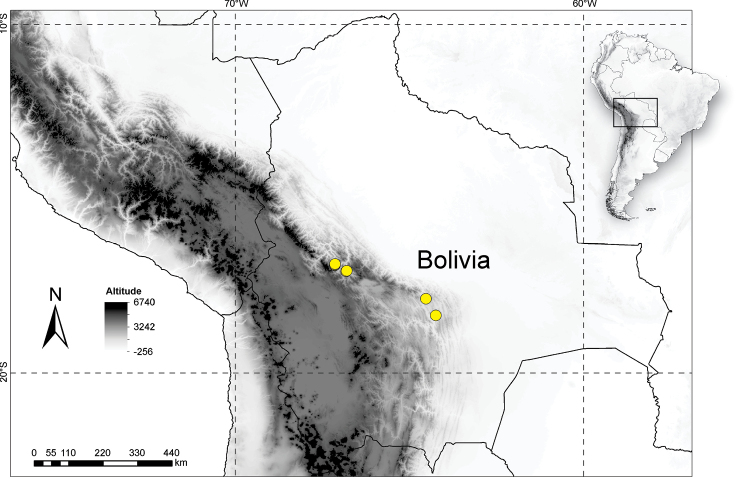
Distribution of *Boliviadendronbolivianum* endemic to the interior valleys on the eastern flanks of the Andes in Bolivia.

The monospecific genus *Boliviadendron* occupies a narrowly restricted distribution endemic to Bolivia and has been recorded from just a small number of localities on the eastern flanks of the Andes at mid-elevations in interior Andean valleys in the Departments of La Paz, Cochabamba and Santa Cruz (Fig. 3).

#### Habitat.

Locally common in fence-lines and remnant patches of subhumid or seasonally dry Yungas forest and secondary thickets of *Baccharisdracunculifolia* DC., *Dasyphyllumbrasiliense* (Spreng.) Cabrera (both Asteraceae), *Dodonaeaviscosa* Jacq. (Sapindaceae) and *Tecomastans* Juss. (Bignoniaceae). Other associated species include *Apurimaciamichelii* Harms., *Coursetiabrachyrhachis* Harms., *Erythrinafalcata* Benth., *Mimosaboliviana* Benth., *M.woodii* Atahuachi & C.E. Hughes, *Parapiptadeniaexcelsa* (Griseb.) Burkart (all Leguminosae), *Schinopsishaenkeana* Engl. (Anacardiaceae), *Cordylinedracaenoides* Kunth (Asparagaceae), *Kageneckialanceolata* Ruiz & Pav. (Rosaceae) and *Cleistocactuslaniceps* (K. Schum.) Rol.-Goss (Cactaceae). *Boliviadendron* is known only from the slopes of interior valleys of Bolivia between 2150 and 2770 m alt., around the transition from seasonally-dry tropical inter-Andean valley forests to more moist mid-elevation montane Ceja de Monte Yungeña vegetation. Most collections (apart from two outlying localities in Dept. Santa Cruz) come from two nearby tributaries of the upper Río Cotacajes, in Prov. Ayopaya, Cochabamba and Prov. Inquisivi, La Paz, Bolivia, an area with several other narrowly-endemic plants, including *Justiciapluriformis* Wash. & J.R.I. Wood (Acanthaceae), *Philibertiafontellae* (Murillo) Goyder (Apocynaceae), *Solanumstellativelutinum* Bitter and *S.tunariense* Kuntze (Solanaceae) and *Mimosawoodii* (Leguminosae). *Boliviadendron* (as *Leucochloronbolivianum*) was assigned an IUCN threat category of Endangered (EN) B1ab(iii,iv) in 2011 (Atahuachi 2012) on account of its very limited extent of occurrence and threats from habitat destruction caused by agricultural expansion and fire which are continuing today.

#### Etymology.

*Boliviadendron* is one of just two mimosoid legume genera known to be endemic to Bolivia (the other being *Pseudosenegalia* Seigler & Ebinger with two species) and that Bolivian endemism is highlighted here by the double-barrelled reference to that country in the name *Boliviadendronbolivianum*.

### ﻿ Boliviadendron bolivianum

Taxon classificationPlantae FabalesLeguminosae

(C.E. Hughes & Atahuachi) E.R. Souza & C.E. Hughes comb. nov.

98D0BD95-6D9A-5C5F-8BD1-B87A765EE40D

urn:lsid:ipni.org:names:77303835-1

#### Basionym.

*Leucochloronbolivianum* C.E. Hughes & Atahuachi, Kew Bull. 61: 559. 2006, publ. 31 Jan 2007.

#### Type.

Bolivia. La Paz, Prov. Inquisivi, 6 km N of Inquisivi, rd towards Cajuata, Circuata and Miguillas, 16°53'42"S, 67°08'23"W, 2385 m alt., 12 Dec 2003, *C.E. Hughes*, *T. Ortuño & M. Mendoza 2423* (holotype: LPB; isotypes: BOLV, FHO, K [K000532854], USZ).

#### Additional material examined.

**Bolivia**. Cochabamba, Prov. Ayopaya, 1 km S of Independencia on rd to Pongo, 17°05'03"S, 66°48'53"W, 2630 m alt., 15 May 2002 (unripe fr.), *C.E. Hughes*, *J.R.I. Wood & R. Forrest 2287* (FHO!, K! [2 specimens], LPB!). Cochabamba, Ayopaya, 1 km above Independencia on road to Sailapata and La Mina, 17°04'S, 66°48'W, 2770 m, 18 Dec 2002 (fl.), *J.R.I. Wood*, *M. Mercado & M. Mendoza 18731* (K!). La Paz, Prov. Inquisivi, ca. 10 km N of Inquisivi, rd to Licoma and Circuata, 16°53'71"S, 67°08'35"W, 2300 m alt., 27 March 2007 (unripe fr.), *C.E. Hughes*, *T. Särkinen*, *A. Wortley & P. Duchen 2608* (FHO!, K! [2 specimens], LPB). La Paz, Prov. Inquisivi, 6 km N of Inquisivi, rd towards Cajuata, 16°53'43"S, 67°08'23"W, 2379 m alt., 12 Feb 2005 (ripe fr.), *J.R.I. Wood*, *M. Atahuachi & T. Ortuño 21607* (BOLV, FHO!, K! [2 specimens], LPB). La Paz, Prov. Inquisivi, 0.5 km above Sica rd from Inquisivi to Cajuata, 16°52'32"S, 67°08'04"W, 2565 m alt., 12 Feb 2005 (fl.), *J.R.I. Wood*, *M. Atahuachi & T. Ortuño 21618* (BOLV, FHO!, K! [2 specimens], LPB). La Paz, Prov. Inquisivi, 13 km from Inquisivi towards Licoma, crossing the Río Quime, 2150 m alt., 24 April 1992 (unripe fr.), *St.G. Beck 21125* (K! [2 specimens]). La Paz, Prov. Inquisivi, along slopes E of Communidad Micayani to the Rio Khokhoni more or less to the junction with a fork flowing down from Communidad Yamora and following the Rio Khokhoni upstream 1 km from this point, ca. 4 km SE from Inquisivi, 16°55'S, 67°06'W, 2650 m alt., 14 Jan 1989 (fl.), *M. Lewis 35094* (K!, LPB, MO). Santa Cruz, Prov. M. Caballero, ascending from Comarapa towards Cerro Bravo, 17°52'25"S, 64°31'32"W, 20 Nov 2005 (fl.), *J.R.I. Wood & M. Mendoza 22081* (BOLV, FHO!, K! [2 specimens], LPB). Santa Cruz, Prov. Valle Grande, 14.2 km on the gravel road towards Moro Moro from the highway in the Trigal / Muyurina valley, 18°21'00"S, 64°14'27"W, 2335 m alt., 1 Jan 2011 (fl.), *M. Nee & J.M. Mendoza F 57498* (NY!).

## Supplementary Material

XML Treatment for Boliviadendron

XML Treatment for Boliviadendron bolivianum

## Supplementary materials

Supplementary material 1 Table S1–S3 https://doi.org/10.3897/phytokeys.205.82256.suppl1 Data type Tables Explanation note Includes voucher information, primers and protocols and summary of phylogenetic analyses. https://binary.pensoft.net/file/733548This dataset is made available under the Open Database License (http://opendatacommons.org/licenses/odbl/1.0/). The Open Database License (ODbL) is a license agreement intended to allow users to freely share, modify, and use this Dataset while maintaining this same freedom for others, provided that the original source and author(s) are credited.Lamarck Rocha

Supplementary material 2 Figures S1–S9 https://doi.org/10.3897/phytokeys.205.82256.suppl2 Data type Individual phylogenetic analyzes Explanation note Includes all trees derived from the BI, MP and ML with combined nuclear + plastidial loci, and individual analyses from nuclear, and plastidial loci. https://binary.pensoft.net/file/733549This dataset is made available under the Open Database License (http://opendatacommons.org/licenses/odbl/1.0/). The Open Database License (ODbL) is a license agreement intended to allow users to freely share, modify, and use this Dataset while maintaining this same freedom for others, provided that the original source and author(s) are credited.Lamarck Rocha
