# Supplementary material for: ﻿Boliviadendron, a new segregate genus of mimosoid legume (Leguminosae, Caesalpinioideae, mimosoid clade) narrowly endemic to the interior Andean valleys of Bolivia
Source: PhytoKeys. 2022 Aug 22;205:439–52. doi: 10.3897/phytokeys.205.82256 (PMC9849042; doi:10.3897/phytokeys.205.82256)
Supplement: Supplementary material 1 — Table S1–S3 [file phytokeys-205-439_article-82256__-s001.docx]

**SUPPLEMENTAL 1**

**Table S1**. Voucher information and GenBank accession numbers for the sequences used in this study. Sequences generated here are presented in **bold**. Missing data = ─.

| **Taxon** | **Voucher (herbarium)** | **Locality** | **GenBank accession numbers** | | | | | | |
| --- | --- | --- | --- | --- | --- | --- | --- | --- | --- |
|  |  |  | **ITS** | **ETS** | ***psbA-trnH*** | ***rps16*** | ***trnD-trnT*** | ***trnL-trnF*** | ***rpL32-trnL*** |
| *Acacia ampliceps* Maslin | D.J. Murphy 323  (MEL) | Australia | AF360718 | EF638117 | AF525003 | ─ | ─ | EU439994 | JQ942520 |
| *Albizia adianthifolia* (Schumach.) W. Wight | BGRO 001 (ITS)  OM1801JRAU (trnL-F) (JRAU) | Africa | MW699934 | ─ | ─ | ─ | ─ | JQ230277 | ─ |
| *Albizia anthelmintica* Brongn. | BGRO 004 (ITS)/OM363 (psbA/trnL-F) (JRAU) | Africa | MW699937 | ─ | JQ230171 | ─ | ─ | JQ230209 | ─ |
| *Albizia polycephala* (Benth.) Killip | K. Almeida 123 (K) | Brazil, Bahia | **KF933275** | **KF921625** | **KF921821** | **KF921781** | **KF921704** | **KF933279** | **KF921867** |
| *Albizia saponaria* (Lour.) Blume | SRA043 (MELU) | Asia | EF638171 | EF638085 | ─ | ─ | ─ | ─ | ─ |
| *Albizia versicolor* Welw. ex Oliv. | RL1214 (JRAU) | Africa | ─ | ─ | JQ230177 | ─ | ─ | JQ230218 | ─ |
| *Blanchetiodendron blanchetii* (Benth.) Barneby & J.W. Grimes | L.P. Queiroz 7085 (HUEFS) | Brazil, Bahia | JX870658 | **KF921626** | **KF921822** | **KF921782** | **KF921705** | JX870790 | **KF921868** |
| ***Boliviadendon bolivianum* (C.E. Hughes & Atahuachi) E.R. Souza & C.E. Hughes** | C.E. Hughes 2423  (FHO) | Bolívia | **KF921699** | **KF921660** | **KF921855** | **KF921809** | **KF921738** | **KF921776** | **KF921901** |
| ***Boliviadendon bolivianum*** | C.E. Hughes 2608  (FHO) | Bolivia | **OM436178** | **─** | **OM456603** | **OM456589** | **OM456596** | **OM456610** | **─** |
| ***Boliviadendon bolivianum*** | J. Wood 21607 (K) | Bolivia | **OM436179** | **─** | **OM456605** | **OM456591** | **OM456598** | **OM456612** | **─** |
| ***Boliviadendon bolivianum*** | Beck 21125 (K) | Bolivia | **OM436176** | **OM456579** | **OM456601** | **OM456587** | **OM456594** | **OM456608** | **OM456582** |
| ***Boliviadendon bolivianum*** | C.E. Hughes 2287 (FHO) | Bolivia | **OM436177** | **OM456580** | **OM456602** | **OM456588** | **OM456595** | **OM456609** | **OM456583** |
| ***Boliviadendon bolivianum*** | C.E. Hughes 2688 (FHO) | Bolivia | **─** | **OM456581** | **OM456604** | **OM456590** | **OM456597** | **OM456611** | **OM456584** |
| ***Boliviadendon bolivianum*** | J. Wood & Mendoza 22081 (K) | Bolivia | **OM436181** | **─** | **OM456606** | **OM456592** | **OM456599** | **OM456613** | **OM456585** |
| *Chloroleucon acacioides* (Ducke) Barneby & J.W. Grimes | A.M. Miranda 5340 (HUEFS) | Brazil, Piauí | **KF921673** | **KF921628** | **KF921824** | **KF921784** | **KF921707** | **KF921751** | **KF921870** |
| *Chloroleucon dumosum* (Benth.) G.P. Lewis | P.G.C. Almeida 7 (HUEFS) | Brazil, Bahia | **KF921676** | **KF921631** | **KF921827** | **KF921786** | **KF921710** | **KF921754** | **KF921873** |
| *Chloroleucon extortum* Barneby & J.W. Grimes | L.P. Queiroz 7287 (HUEFS) | Brazil, Bahia | **KF921681** | **KF921636** | **KF921832** | **KF921789** | **KF921715** | **KF921758** | **KF921878** |
| *Chloroleucon foliolosum* (Benth.) G.P. Lewis | D. Cardoso 134 (HUEFS) | Brazil, Bahia | **KF921683** | **KF921638** | **KF921834** | **KF921790** | **KF921717** | **KF921761** | **KF921880** |
| *Chloroleucon mangense* Britton & Rose var. mangense | A. Reyes-Garcia 5795 (HUEFS) | México | **KF921690** | **KF921645** | **KF921841** | **KF921796** | **KF921724** | **KF921768** | **KF921887** |
| *Chloroleucon mangense* var. lentiscifolium (A. Rich.) Barneby & J.W. Grimes | E. Martinez 5/37281 (MBM) | México | **KF921689** | **KF921644** | **KF921840** | ─ | **KF921723** | **KF921767** | **KF921886** |
| *Chloroleucon tenuiflorum* (Benth.) Barneby & J.W. Grimes | C.E. Hughes 2450 (FHO) | Bolívia, Cochabamba | **KF921691** | **KF921646** | **KF921842** | **KF921797** | **KF921725** | **KF921769** | **KF921888** |
| *Chloroleucon tortum* (Mart.) Barneby & J.W. Grimes | E.R. Souza 940 (HUEFS) | Brazil, Rio de Janeiro | **KF921694** | **KF921649** | **KF921845** | **KF921798** | **KF921727** | **KF921771** | **KF921890** |
| *Ebenopsis confinis* Britton & Rose | M. Souza 224 (MEXU) | Mexico | **KF921695** | **KF921650** | **KF921846** | **KF921799** | **KF921728** | **KF921772** | **KF921891** |
| *Ebenopsis ebano* (Berland.) Barneby & J.W. Grimes | Q.B.A. Feliciano 358 (MEXU) | Mexico | **JX870759** | **KF921651** | **KF921847** | **KF921800** | **KF921729** | **JX870875** | **KF921892** |
| *Enterolobium gummiferum* J.F.Macbr. | J.D. Peixinho 31832 (MEXU) | Brazil, Bahia | **KF921696** | **KF921652** | **KF921848** | **KF921801** | **KF921729** | **KF921773** | **KF921893** |
| *Enterolobium schomburgkii* Benth. | L.P. Queiroz 13931 (HUEFS) | Brazil, Amazonas | **KF921697** | **KF921653** | **KF921849** | **KF921802** | **KF921731** | **KF921774** | **KF921894** |
| *Enterolobium timbouva* Mart. | L.P. Queiroz 7973 (HUEFS) | Brazil, Bahia | **JX870760** | **KF921654** | **KF921850** | **KF921803** | **KF921732** | **JX870876** | **KF921895** |
| *Havardia mexicana* Britton & Rose | G.A.L. Reina 98528 (MEXU) | Mexico | **KF933276** | **KF921655** | **KF921851** | **KF921804** | **KF921733** | **KF933280** | **KF921896** |
| *Havardia pallens* (Benth.) Britton & Rose | R.H. Magaña 6462 (MEXU) | Mexico | **KF921698** | **KF921656** | **KF921852** | **KF921805** | **KF921734** | **KF921775** | **KF921897** |
| *Hydrochorea corymbosa* (Rich.) Barneby & J.W. Grimes | G.C. Ferreira 571 (K) | Brazil, Pará | JX870763 | **KF921657** | ─ | **KF921806** | **KF921735** | **JX870879** | **KF921898** |
| *Inga edulis* Mart. | L.P. Queiroz 13797 (HUEFS) | Brazil, Bahia | JX870764 | **KF921658** | **KF921853** | **KF921807** | **KF921736** | **JX870880** | **KF921899** |
| *Inga thibaudiana* DC. | M.A. Costa 1001 (HUEFS) | Brazil, Manaus | JX870765 | **KF921659** | **KF921854** | **KF921808** | **KF921737** | **JX870881** | **KF921900** |
| *Jupunba piresii* (Barneby & J.W. Grimes) M.V.B.Soares, M.P.Morim & Iganci | P.A.C.L. Assunção 411 (SP) | Brazil, Amazonas | JX870655 | **KF921624** | **KF921820** | **KF921780** | **KF921703** | **KF933278** | **KF921866** |
| *Leucochloron foederale* (Barneby & J.W. Grimes) Barneby & J.W. Grimes | L.V. Costa s.n. (HUEFS 35816) | Brazil, Minas Gerais | **KF921700** | **KF921661** | **KF921856** | **KF921810** | **KF921739** | **KF921777** | ─ |
| *Leucochloron incuriale* (Vell.) Barneby & J.W. Grimes | F.M. Ferreira 1978 (HUEFS) | Brazil, Minas Gerais | **KF921701** | **KF921662** | **KF921857** | **KF921811** | **KF921740** | **KF921778** | **KF921902** |
| *Leucochloron limae* Barneby & J.W. Grimes | A.M. Carvalho 5339 (K, DNA Bank sample) | Brazil, Bahia | JX870766 | **KF921663** | **KF921858** | **KF921811** | **KF921741** | **JX870882** | **KF921903** |
| *Leucochloron minarum* (Glaz. ex Harms) Barneby & J.W. Grimes | J.M. Fernandes 1415 (VIC) | Brazil, Minas Gerais | **KF921702** | **KF921664** | **KF921859** | **KF921813** | **KF921742** | **KF921779** | **KF921904** |
| *Lysiloma acapulcense* (Kunth) Benth. | D. Seigler 15988 (ILL) | Mexico | ─ | EF638092 | AF524977 | ─ | ─ | AF522958 | ─ |
| *Lysiloma divaricatum* (Jacq.) Benth. | D. Seigler 15994 (ILL) | Mexico | ─ | EF638094 | ─ | ─ | ─ | AF522940 | ─ |
| *Lysiloma latisiliquum* (L.) Benth. | C.E. Hughes 486 (FHO) | México | **KF933277** | ─ | ─ | ─ | **KF938591** | **KF933281** | ─ |
| *Macrosamanea pubiramea* (Steud.) Barneby & J.W. Grimes | J.G. Jardim 4595 (HUEFS) | Venezuela, Amazonas | JX870767 | **KF921665** | **KF921860** | **KF921814** | **KF921743** | JX870883 | **KF921905** |
| *Pithecellobium diversifolium* Benth. | L.P. Queiroz 3740 (K) | Brazil, Bahia | JX870768 | **KF921666** | ─ | **KF921815** | **KF921744** | JX870884 | **KF921906** |
| *Pseudosamanea guachapale* (Kunth) Harms | J.E. Madsen 83914 (K) | Ecuador, Guayas | JX870769 | **KF921667** | **KF921861** | **KF921816** | **KF921745** | JX870885 | **KF921907** |
| *Samanea saman* (Jacq.) Merr. | E.R. Souza 386 (HUEFS) | Brazil, Bahia | JX870770 | **KF921668** | **KF921862** | **KF921817** | **KF921746** | JX870886 | **KF921908** |
| *Sphinga acatlensis* (Benth.) Barneby & J.W.Grimes | A.B. Martinez 339 (MEXU) | Mexico | JX870771 | **KF921669** | **KF921863** | **KF921818** | **KF921747** | JX870887 | **KF921909** |
| *Thailentadopsis nitida* (Vahl) G.P. Lewis & Schrire | A. Kostermans 28234 (K) | Sri Lanka | JX870772 | **KF921670** | **KF921864** | **─** | **KF921748** | JX870888 | **─** |
| *Zygia racemosa* (Ducke) Barneby & J.W. Grimes | J.E.L.S. Ribeiro 1387 (SP) | Brazil, Manaus | JX870785 | **KF921671** | **KF921865** | **KF921819** | **KF921749** | JX870900 | **KF921910** |

**Table S2**. Primers and protocols used for PCR and best-fitting substitution models for each partition based on AIC.

| **Region** | **Primers** | **Protocol** | **Model** | **Notes** |
| --- | --- | --- | --- | --- |
| **ITS** | ITS-17SE (F) 5'-ACG AAT TCA TGG TCC GGT GAA GTG TTC G-3'  ITS-26SE (R) 5'-TAG AAT TCC CCG GTT CGC TCG CCG TTA C-3'  ITS-92 (F) 5'-AAG GTT TCC GTA GGT GAA C-3'  ITS-4 (R) 5'-TCC TCC GCT TAT TGA TAT GC-3' | 94 °C for 3 min, 28–30 × (96 °C for 45 sec, 54–56 °C for 1 min, 72 °C for 1 min), 72 °C for 7 min | ITS1/ITS2: GTR + I + Γ.  5.8S: K80. | 17SE and 26SE (Sun et al. 1994); ITS92 (Desfeaux et al. 1996) and ITS4 (White et al. 1990) |
| **ETS** | ETS-B(F) 5'-ATA GAG CGC GTG AGT GGT G-3'  AcR_2_(R) 5'-GGG CGT GTG AGT GGT GTT TGG-3' | 94 °C for 3 min, 30 × (96 °C for 1 min, 54–56 °C for 1 min, 72 °C for 2 min), 72 °C for 7 min | GTR + Γ | AcR2 (Ariati et al. 2006) and 18S-IGS (Baldwin and Markos, 1998) |
| ***psbA-trnH*** | PsbA(F) 5'-GTT ATG CAT GAA CGT AAT GCT C-3'  TrnH(R) 5'-CGC GCA TGG TGG ATT CAC AAA TC-3' | 94 °C for 1 min, 40 × (94 °C for 30 sec, 53 °C for 40 sec, 72 °C for 40 sec), 72 °C for 5 min | GTR + Γ | Aldrich et al. 1988 |
| ***rps16*** | rps16(F) 5'-GTG GTA GAA AGC AAC GTG CGA CTT-3'  rps16(R) 5'-TCG GGA TCG AAC ATC AAT TGC AAC-3' | 94 °C for 2 min, 35 × (94 °C for 1 min, 53–55 °C for 1 min, 72 °C for 2 min), 72 °C for 3 min | GTR + I + Γ | Oxelman et al. 1997 |
| ***rpl32*** | rpl32(F) 5'-CAG TTC CAA AAA AAC GTA CTT C-3'  rpl32(R) 5'-CAG TTC CAA AAA AAC GTA CTT C-3' | 80 °C for 5 min, 30 × (95 °C for 1 min, 55 °C for 1 min, 65 °C for 4 min), 65 °C for 4 min | GTR + I + Γ | Shaw et al. 2007 |
| ***trnD-T*** | trnD(F) 5'-ACC AAT TGA ACT ACA ATC CC-3'  trnE(R) 5'-AGG ACA TCT CTC TTT CAA GGA G-3'  trnY(F) 5'-CCG AGC TGG ATT TGA ACC A-3'  trnT(R) 5'-CTA CCA CTG AGT TAA AAG GG-3' | 94°C for 5 min, 36 × (94°C for 50 seg, 54°C for 50 seg, 72°C for 1,30 min), 72°C for 5 min | GTR + I + Γ | t*rnE* and *trnY* (Shaw et al. 2005); *trnD* and *trnT* (Demesure et al. 1995) |
| ***trnL-intron*** | trnL-C(F) 5'-CGA AAT CGG TAG ACG CTA CG-3'  trnL-D(R) 5'-GGG GAT AGA GGG ACT TGA AC-3' | 94 °C for 3 min, 35 × (94 °C for 1 min, 56 °C for 1 min, 72 °C for 2 min), 72 °C for 10 min | GTR + Γ | Taberlet et al. 1991 |
| ***trnL-F*** | trnL-E(F) 5'-GGT TCA AGT CCC TCT ATC CC-3'  trnL-F(F) 5'-ATT TGA ACT GGT GAC ACG AG-3' | 94°C for 2 min, 35 × (94°C for 50 seg, 60°C for 25 seg, 72°C for 1,25 min), 72°C for 3 min | GTR + I + Γ | Taberlet et al. 1991 |

**Table S3**. Summary of phylogenetic analyses. Align. = length of the aligned molecular matrix; PI = number of parsimony informative characters; CI = consistency index; RI = retention index.

| **Dataset** | **Align. (bp)** | **Variable characters** | **PI** | **MP tree length** | **CI** | **RI** |
| --- | --- | --- | --- | --- | --- | --- |
| ITS | 894 | 120 | 151 | 677 | 0.5377 | 0.7208 |
| ETS | 540 | 100 | 193 | 752 | 0.5479 | 0.7314 |
| *psbA-trnH* | 529 | 95 | 72 | 286 | 0.6853 | 0.7134 |
| *rps16* | 997 | 102 | 55 | 221 | 0.8145 | 0.7405 |
| *rpl32* | 725 | 117 | 78 | 322 | 0.7112 | 0.7249 |
| *trnD-T* | 1544 | 165 | 72 | 320 | 0.7906 | 0.7607 |
| *trnL intron* + *trnL-F* | 1197 | 134 | 90 | 323 | 0.7430 | 0.7036 |
| Plastidial | 4992 | 613 | 367 | 1633 | 0.6724 | 0.6095 |
| Nuclear | 1434 | 220 | 344 | 1495 | 0.5191 | 0.6988 |
| Pastidial+ Nuclear | 6426 | 833 | 711 | 3200 | 0.5856 | 0.6471 |

**References for Table S1**

Aldrich J, Cherney BW, Merlin E, Christopherson L (1988) The role of insertions/deletions in the evolution of the intergenic region between psbA and trnH in the chloroplast genome. Current Genetics 14: 137–146. https://10.1007/bf00569337

Ariati SR, Murphy DJ, Udovicic F, Ladiges PY (2006) Molecular phylogeny of three groups of acacias (*Acacia* subgenus *Phyllodineae*) in arid Australia based on the internal and external transcribed spacer regions of nrDNA. Systematics and Biodiversity 4: 417–426. https://doi.org/10.1073/pnas.1100628108

Baldwin BG, Markos S (1998) Phylogenetic Utility of the External Transcribed Spacer (ETS) of 18S-26S rDNA: Congruence of ETS & ITS trees of *Calycadenia* (Compositae). Molecular Phylogenetics and Evolution 10: 449–463. https://doi.org/10.1006/mpev.1998.0545

Demesure B, Sodzi N, Petit RJ (1995) A set of universal primers for amplification of polymorphic non-coding regions of mitochondrial and chloroplast DNA in plants. Molecular Ecology 4: 129–131. https://10.1111/j.1365-294x.1995.tb00201.x

Desfeaux C, Maurice S, Henry JP, Lejeune B, Gouyon PH (1996) The evolution of reproductive systems in the genus *Silene*. Proc. of the Royal Soc. Of London 263: 409–414. https://10.1098/rspb.1996.0062

Oxelman B, Lidén M, Berglund D (1997) Chloroplast rps16 intron phylogeny of the tribe *Sileneae* (Caryophyllaceae). Plant Systematics and Evolution 206: 393–410. https://doi.org/10.1007/BF00987959

Shaw J, Lickey EB, Beck JT, Farmer SB, Liu W, Miller J, Siripun KC, Winder CT, Schilling EE, Small RL (2005) The tortoise and the hare II: relative utility of 21 noncoding chloroplast DNA sequences for phylogenetic analysis. American Journal of Botany 92: 142–166. https://doi.org/10.3732/ajb.92.1.142

Shaw J, Lickey EB, Schilling EE, Small RL (2007) Comparison of whole chloroplast genome sequences to choose non-coding regions for phylogenetic studies in angiosperms: the tortoise and the hare III. American Journal of Botany 94: 275–288. https://doi.org/10.3732/ajb.94.3.275

Sun Y, Skinner DZ, Liang GH, Hulbert SH (1994) Phylogenetic analysis of *Sorghum* and related taxa using internal transcribed spacer ribosomal DNA. Theoretical and Applied Genetics 89: 26–32. https://doi.org/10.1007/BF00226978

Taberlet P, Gielly L, Pautou G, Bouvet J (1991) Universal primers for amplifications of three non-coding regions of chloroplast DNA. Plant Molecular Biology 17: 1105–1109.

White TJ, Bruns T, Lee S, Taylor J (1990) Amplification and direct sequencing of fungal ribosomal RNA genes for phylogenetics. In: Innis M, Gelfand D, Sninsky J, White T (Eds.) PCR Protocols: A Guide to Methods and Applications. Academic Press, California, 315–322.
