# Supplementary material for: ﻿Boliviadendron, a new segregate genus of mimosoid legume (Leguminosae, Caesalpinioideae, mimosoid clade) narrowly endemic to the interior Andean valleys of Bolivia
Source: PhytoKeys. 2022 Aug 22;205:439–52. doi: 10.3897/phytokeys.205.82256 (PMC9849042; doi:10.3897/phytokeys.205.82256)
Supplement: Supplementary material 2 — Figures S1–S9 [file phytokeys-205-439_article-82256__-s002.docx]

**SUPPLEMENTAL 2**

**Figure S1**. Majority-rule (50%) consensus tree derived from the BI combined analysis of nuclear (ITS and ETS) and plastid (*psbA, rpL32-trnL, rps16, trnD-T, trnL* and *trnL-F*) DNA. Posterior Probabilities (PP) support values beside the nodes.





**Figure S2**. Majority-rule (50%) consensus tree from MP combined analysis of nuclear (ITS an ETS) and plastid (*psbA, rpL32-trnL, rps16, trnD-T, trnL* and *trnL-F*) DNA. Bootstrap (BS) support values beside the nodes.





**Figure S3**. ML tree from combined analysis of nuclear (ITS and ETS) and plastid (*psbA, rpL32-trnL, rps16, trnD-T, trnL* and *trnL-F*) DNA. Bootstrap (BS) support values beside the nodes.





**Figure S4**. Majority-rule (50%) consensus tree derived from the BI analysis of ITS and ETS. Posterior Probabilities (PP) support values beside the nodes.





**Figure S5**. Majority-rule (50%) consensus tree from MP analysis of ITS and ETS. Bootstrap (BS) support values beside the nodes.





**Figure S6**. ML tree from analysis of ITS and ETS. Bootstrap (BS) support values beside the nodes.





**Figure S7**. Majority-rule (50%) consensus tree derived from the BI analysis of *psbA, rpL32-trnL, rps16, trnD-T, trnL* and *trnL-F*. Posterior Probabilities (PP) support values beside the nodes.





**Figure S8**. Majority-rule (50%) consensus tree from MP analysis of *psbA, rpL32-trnL, rps16, trnD-T, trnL* and *trnL-F*. Bootstrap (BS) support values beside the nodes.





**Figure S9**. ML tree from analysis of *psbA, rpL32-trnL, rps16, trnD-T, trnL* and *trnL-F*. Bootstrap (BS) support values beside the nodes.
